# Sugar Sweetened and Artificially Sweetened Beverage Consumption and Pancreatic Cancer: A Retrospective Study

**DOI:** 10.3390/nu15020275

**Published:** 2023-01-05

**Authors:** Evan W. Davis, Susan E. McCann, Janine M. Joseph, Karen H. K. Yeary, Christos Fountzilas, Kirsten B. Moysich

**Affiliations:** 1 Department of Cancer Prevention and Control, Roswell Park Comprehensive Cancer Center, Buffalo, NY 14263, USA; 2 Division of GI Medicine, Department of Medicine, Roswell Park Comprehensive Cancer Center, Buffalo, NY 14263, USA

**Keywords:** pancreatic cancer, sugar sweetened beverages, artificially sweetened beverages, diet, lifestyle factors, risk, mortality

## Abstract

Pancreatic cancer (PanCa) is a highly fatal malignancy with few modifiable risk and prognostic factors. This study investigates the association between cola, diet cola, and non-cola soft drink consumption and PanCa risk and mortality. A retrospective study was conducted using data from the Patient Epidemiology Data System (1982–1998) at Roswell Park Comprehensive Cancer Center (Buffalo, NY, USA), including 213 PanCa patients and 852 cancer-free controls. Data were collected using a self-administered questionnaire, including a 46-item food frequency questionnaire (FFQ). Multivariable logistic regression was used to estimate odds ratio (OR) and 95% confidence interval (CI) of cola, diet cola, and non-cola soft drink consumption and PanCa risk. Cox proportional hazard regression was used to estimate hazard ratios (HR) and 95% CIs of cola, diet cola, and non-cola soft drink consumption and PanCa mortality. Stratified analyses were conducted by sex, body mass index (BMI), and smoking status. We observed significant 55% increased odds of PanCa among patients consuming ≥1 regular cola per day (OR: 1.55, 95% CI: 1.01–2.39). We also observed non-significant 38% increased hazard of mortality among patients consuming ≥1 regular cola per day (HR: 1.38, 95% CI: 0.91–2.07). We conclude that regular cola consumption is a modifiable lifestyle that may be associated with PanCa risk and mortality following diagnosis.

## 1. Introduction

In the US, pancreatic cancer (PanCa) is the 10th and 8th most common cancer in men and women, respectively, and the 4th most deadly among both sexes with incidence increasing since 2000 [1]. PanCa-related symptoms become apparent only in advanced stages, and the 5-year survival rate has made only modest improvements over the last 40 years and remains poor at approximately 10.8%, despite improved surgical skills and treatment modalities [1]. Identifying modifiable risk and prognostic factors is critical due to the poor prognosis of this disease. Cigarette smoking, obesity, type 2 diabetes, chronic pancreatitis, and family history of PanCa are well-established risk factors [1,2,3,4]. More research is needed to assess other potential modifiable risk factors that may be associated with PanCa development. There are currently no known modifiable prognostic factors for PanCa warranting further investigation in this line of research.

One modifiable dietary risk factor is the consumption of sweetened beverages including both sugar sweetened beverages (SSB), beverages sweetened with a naturally derived sweetener, and artificially sweetened beverages (ASB), beverages sweetened with manufactured sweeteners such as aspartame. Higher consumption of added dietary sugars (sucrose, fructose, and high fructose corn syrup) has been associated with increased weight gain [5], obesity [6], and type 2 diabetes [7], which are known risk factors of PanCa [8,9]. These health conditions give rise to insulin resistance [10], oxidative stress [11], and chronic inflammation [11], all conditions clearly linked to cancer development and progression of cancer [12,13,14]. Insulin resistance increases the production of insulin and insulin-like growth factors that are associated with increased cellular proliferation and survival posing a likely oncogenic role [12]. Additionally, recent work has concluded that artificial sweetener (aspartame and acesulfame-K) consumption may be associated with increased risk of developing an obesity-related cancer, such as PanCa [15,16].

With the mounting biological evidence for the association of SSB and ASB consumption and cancer, numerous epidemiologic studies revealed positive associations between SSB and ASB consumption and the development of breast, prostate, colorectal, and PanCa cancers; however, only the associations for breast and prostate cancers reached statistical significance [17]. The associations between SSB and ASB consumption and PanCa risk are inconsistent [18,19,20,21,22,23,24,25,26,27,28]. A recent meta-analysis evaluating SSB and ASB consumption separately reported a non-significant positive association with higher consumption of SSBs and ASBs and PanCa [17]. A gap remains to further study PanCa with a more granular analysis within SSB and ASB categories to determine the source of the increased risk within the categories. Additionally, higher SSB and ASB consumption patterns in upper aerodigestive cancers may decrease survival [29]. That, in conjunction with the biological evidence for the role of SSB and ASB consumption in cancer progression, warrants further investigation into their roles in PanCa mortality. To our knowledge, no such study has been performed in PanCa.

According to a 2017 report, almost two-thirds of youth and about half of adults in the US consumed at least one SSB on any given day between 2011 and 2014 [30]. While recent trends in heavy SSB consumption are declining among youth and adult populations [31], there remain many individuals whose habitual consumption of SSBs may be increasing their risk of PanCa and poor PanCa outcomes. This study aims to elucidate the association between SSB and ASB consumption and PanCa risk by expanding on previous work to include additional obesity-related factors such as diet quality as well as assessing the role of SSBs and ASBs separately. Additionally, we aim to evaluate SSB and ASB consumption as a prognostic factor for PanCa.

## 2. Materials and Methods

### 2.1. Study Population

We conducted a hospital-based case–control study in patients evaluated for suspected malignancy at Roswell Park Comprehensive Cancer Center between 1982 and 1998 and participated in the Patient Epidemiologic Data System (PEDS). PEDS data were last collected in 1998 and represent the largest dataset of PanCa cases at Roswell Park with detailed SSB and ASB consumption data. Patients were offered an epidemiologic questionnaire during the admission process to collect data on demographics, lifestyle habits, medical history, diet, and other epidemiologic variables. Approximately 50% of patients completed this instrument. Participants in this study included 213 patients with PanCa and 852 patients with non-cancer diagnoses. The controls were frequency matched to cases by sex, five-year categories of age, and five-year categories of year the survey was completed to control for population-wide changes in risk factors throughout the data collection period. The patient population was predominately White (97%) and between the ages of 30 and 89 years at the time of diagnosis.

Additionally, we conducted a retrospective cohort study using the 213 PanCa cases identified from the PEDS data in our case–control study to examine the associations of SSB and ASB consumption and mortality. Only 10 patients survived the duration of the follow-up due to the low survival rate of PanCa. Thus, for our descriptive analyses, we divided the PanCa patients into two groups: those who survived 1 year or less (*n* = 133) and those who survived more than 1 year after diagnosis (*n* = 80). Clinical follow-up data including vital status and date of last contact were obtained from the Cancer Registry at Roswell Park. As a part of the PEDS questionnaire, each participant completed a 46-item food frequency questionnaire (FFQ) querying information about general dietary habits in the few years prior to diagnosis including daily consumption of SSB as regular cola and regular non-cola soft drinks and daily consumption of ASB as diet cola. The SSB and ASB consumption data were each grouped into three categories: “Never Consumers”, “Occasional Consumers”, and “Habitual Consumers”. More information regarding SSB and ASB classification can be found in Supplementary Materials Tables S1 and S2.

### 2.2. Statistical Analysis

Characteristics of the cases and controls were compared using Pearson’s chi-square or Fisher Exact test for categorical variables where indicated and Student’s *t*-test for continuous variables. Odds ratios (OR) and 95% confidence intervals (CI) were estimated using multivariable logistic regression to evaluate the association between all regular soft drinks, all cola drinks, regular cola, diet cola, and regular non-cola soft drink consumption, referent to non-consumers, and PanCa. The associations were adjusted for age, sex, smoking status, body mass index (BMI) categories, total weekly vegetable intake, weekly processed meat intake, and family history of PanCa. Covariables were included in the multivariable model if they differed significantly (*p* ≤ 0.10) between the cases and controls, if their inclusion altered the crude association by 10% or more, or if there was an a priori reason based on biological evidence or previous literature. Sub-group analyses were determined by a priori reasoning based on biological evidence or previous literature or if there was a statistically significant interaction between the variables (*p* ≤ 0.10) and were conducted according to smoking status, BMI categories (Normal Weight (BMI = 18.5–24.9 kg/m^2^) and Overweight/Obese (BMI ≥ 25.0 kg/m^2^)), and sex.

For mortality, we compared characteristics of patients who survived 1 year or less to patients who survived more than 1 year after diagnosis using Pearson’s chi-square for categorical variables and Student’s *t*-test for continuous variables except for median survival time. Cox proportional hazard regression was used to estimate hazard ratios (HR) and corresponding 95% CIs to evaluate the association between each beverage, referent to non-consumers, and mortality following PanCa diagnosis. Covariables were considered for inclusion as described above. The final multivariable model included age, sex, smoking status, BMI, stage at diagnosis, histology, surgery status, radiation status, and total weekly vegetable intake. Sub-group analyses were conducted according to smoking status, BMI categories, and sex as described above but their results are not reported due to the associations being underpowered. All analyses were performed using SAS for Windows, version 9.4 (SAS Institute Inc., Cary, NC, USA). All tests were two-sided and *p*-values ≤0.05 were considered statistically significant.

## 3. Results

### 3.1. Risk

Descriptive characteristics of the study participants are detailed in Table 1. We observed no statistically significant differences between cases and controls for participant characteristics except for family history of PanCa and race. Cases were more likely to have a family history of PanCa (5.2% vs. 2.6%, cases vs. controls, *p* = 0.05) and White race (97.2% vs. 93.2%, cases vs. controls, *p* = 0.03). However, the number of non-White cases was small (*n* = 6).

Multivariable-adjusted OR and 95% CIs for associations between PanCa and SSB and ASB consumption are detailed in Table 2. Compared to non-consumers, we observed a statistically significant association between habitual regular cola consumption and PanCa (OR: 1.55, 95% CI: 1.01, 2.39). No associations were observed for all other beverages.

In the stratified analyses, we observed a statistically significant association between occasional, regular cola consumption, referent to no consumption, and PanCa among overweight/obese (BMI ≥25 kg/m^2^) participants (OR: 1.71, 95% CI: 1.06, 2.76). We also observed a significant association between habitual, diet cola consumption and PanCa among current smokers (OR: 3.34, 95% CI: 1.12, 9.98). All other stratified analyses showed no statistically significant associations. Results of the stratified analyses are detailed in the supplemental materials (Supplementary Materials Table S3).

### 3.2. Mortality

Descriptive characteristics of PanCa patients are detailed in Table 3. Median survival time was 6.1 months for patients who survived 1 year or less and 23.7 months for patients who survived greater than 1 year. Patients who survived 1 year or less consumed fewer servings of fruits (10.3 mean Servings Per Week (SPW)) vs. 12.6 mean SPW, ≤1 year vs. >1 year, *p* = 0.05) and vegetables (17.7 mean SPW versus 20.5 mean SPW, ≤1 year vs. >1 year, *p* = 0.03) each week, had distant stage disease (68.4% vs. 42.5%, ≤1 year vs. >1 year, *p* = 0.001), had adenocarcinoma histology (91.0% vs. 67.5%, ≤1 year vs. >1 year, *p* < 0.0001), received no surgery (13.5% vs. 43.8%, ≤1 year vs. >1 year, *p* < 0.0001) or radiation (29.3% vs. 53.8%, ≤1 year vs. >1 year, *p* < 0.0001), and were White (99.3% versus 93.8%, ≤1 year vs. >1 year, *p* = 0.02). As previously stated, the number of non-White cases was small and precludes any conclusions being drawn regarding race. All other patient characteristics had no statistically significant differences with respect to survival time.

Multivariable-adjusted HR and 95% CIs for the association between SSB and ASB consumption and mortality after PanCa diagnosis are detailed in Table 4. Habitual regular cola consumers had a borderline significant age-adjusted association with PanCa mortality (HR: 1.43, 95% CI: 0.99, 2.07) that was attenuated in the multivariable-adjusted model (HR: 1.38, 95% CI: 0.91, 2.07). No associations were observed for all other beverages.

## 4. Discussion

Our findings contribute to the growing evidence surrounding SSB and ASB consumption and PanCa risk and mortality suggesting that higher patterns of consumption in the few years prior to diagnosis may lead to increased risk of developing PanCa and its subsequent mortality. First, referent to non-consumers, people who consumed at least one regular cola per day were at an increased odds of developing PanCa. When stratified by smoking status, associations with PanCa were strengthened among overweight/obese people consuming regular cola and current smokers consuming diet cola. Second, in our exploratory analysis of PanCa mortality, patients who consumed at least one regular cola per day may be at an increased hazard of mortality compared to those who never consumed regular cola in the few years prior to diagnosis.

Contrary to previous reports on the association of regular cola consumption and PanCa [20,27], we observed a statistically significant positive association with habitual regular cola consumption. Previous studies were limited by the referent group being those who consumed less than one regular cola per month as opposed to our study which used no consumption in the years prior to diagnosis as the referent group [20,27]. Additionally, the consumption pattern groupings in Schernhammer et al. were categorized more broadly with the highest consumption group being greater than three regular colas per week [27] as opposed to our study where the highest consumption group was one or more per day. Finally, our study population had approximately 21% more cases who reported consuming at least one regular cola per day (*n* = 46) than Chan et al. (*n* = 38) [20]. Other reports on SSBs that did not conduct separate analyses for regular cola showed statistically significant and non-statistically significant positive associations [18,22,23,25]. These observations could be due to the use of the catch-all SSB category including both cola and non-cola beverages. Our findings corroborate this hypothesis. All SSBs combined had a non-statistically significant positive association, while regular cola consumption had a statistically significant positive association and non-cola soft drinks had no association. These findings suggest that all SSBs combined in other studies may show a positive association due to the inclusion of regular cola.

Though our findings for the association between SSB and ASB consumption and PanCa mortality did not reach statistical significance, this is the first study to our knowledge to examine SSB and ASB consumption as a potential modifiable prognostic factor for PanCa. Our findings are consistent with one other study that examined the association between SSB and ASB consumption and mortality in upper aerodigestive cancers [29]. Though potential mechanisms are understudied, we hypothesize that SSB and ASB consumption in the few years prior to diagnosis may impact survival through its effects on the accumulation and distribution of adiposity [32]. Accumulation and distribution of adiposity leading to a state of obesity after diagnosis may contribute to a proinflammatory tumor microenvironment which is known to negatively impact PanCa survival [33,34]. Further investigation with a larger sample is needed.

Our sub-group analyses suggest an increased risk of PanCa among overweight/obese patients who consumed regular cola in the years leading up to diagnosis, thus speaking to potential mechanisms of regular cola’s contribution to PanCa risk. SSB consumption can lead to one becoming overweight/obese, which is a known risk factor for PanCa [1,35]. Thus, consumption of regular cola may be causing a type of adiposity that is uniquely favorable to the promotion of PanCa. These findings further support the role of obesity in PanCa risk [1,2,8] especially regarding its relation to SSB consumption [6,10,11] and the contribution of obesity-related inflammation altering the tumor microenvironment [36], which could also be affecting PanCa mortality [37]. Additionally, our sub-group analyses suggested an increased risk of PanCa among current smokers who consumed diet cola in the years leading to diagnosis. Previous work has suggested that increased SSB/ASB consumption may be associated with smoking status with those consuming higher amounts of those beverages being current smokers [38,39,40]. To this end, our observed association may be the result of a synergistic effect between smoking and diet cola consumption both contributing to the risk of PanCa through their respective mechanisms since there is a suggestion of them being correlated [15,38,39,40,41]. Additionally, smoking has been found to increase insulin resistance in patients with diabetes [42,43], which is a known risk factor for PanCa. As previously stated, we do not have adequate data on diabetes status and were thus unable to examine its effects on these associations. Diabetes could represent an uncontrolled confounder or effect modifier that should be examined in future studies.

Our principal observations allow us to deduce a hypothesis for the potential mechanism of the observed associations. Sugar intake alone does not entirely explain the mechanism of the associations between regular cola and PanCa risk and mortality as we did not observe associations with non-cola soft drinks. Cola beverages contain phosphoric acid, which could be contributing to the mechanism. Habitual consumption of phosphoric acid-containing cola products in place of products that are higher in calcium results in reduced dietary consumption of calcium [44]. Calcium plays a crucial role in pancreatic physiology and pathology [45] and, according to Zablotska et al. low dietary consumption of calcium may be associated with PanCa risk [46]. Since regular cola is the only beverage in the present study that contains both sugar and phosphoric acid, we hypothesize that a synergistic effect may exist between phosphoric acid and sugar that contributes to the development and progression of PanCa. The potential role of increased sugar consumption in carcinogenesis is well established biologically [5,6,7,8,9,10,11,12,13,14], but the effect of phosphoric acid and its potential synergistic effect with sugar needs further exploration.

One of our limitations was the inability to examine the role of diabetes in our analyses. Diabetes status was not collected until year 13- of the 17-year data collection period and data were collected prior to electronic medical records systems. Diabetes is likely an uncontrolled confounder of the associations since it is a risk factor for PanCa and SSB/ASB consumption contributes to diabetes development [3,4,7]. Another limitation is the lack of knowledge of the participant’s history of chronic pancreatitis as it is an important risk factor of PanCa [1,2] and may be an effect modifier of the observed associations. Other limitations include a small sample size limiting our stratified risk and survival analyses due to low statistical power and a lack of generalizability due to the data being collected between 1982 and 1998. Additionally, our study population was primarily White, preventing generalization of the findings to underserved groups that bear a disproportionate burden of both SSB intake and PanCa outcomes, particularly Black people who have the highest SSB consumption and highest incidence of PanCa across racial groups [47,48,49,50,51]. Though American Indian/Alaska Natives have a higher PanCa incidence than their White counterparts, they represent a small subset of the overall population, limiting their impact of the associations [51]. In contrast, Hispanic people represent a substantial percentage of the US population but have a lower PanCa incidence and higher SSB consumption patterns than White people [47,48,49,51]. To this end, the potential public health impact could be expected to be greatest among Black people due to higher exposure to SSBs and higher incidence of PanCa, but could be significant among Hispanic people considering their high prevalence of SSB consumption and high percentage of the US population. The full public health implications of the associations observed in our study are likely to be higher in a more racially diverse patient population, but additional studies in more diverse populations are needed. There is also a risk of recall bias with the use of a self-reported FFQ. This may have led to differential misclassification of SSB and ASB consumption patterns with PanCa cases more likely to have heightened recollection of their consumption habits. Additionally, the FFQ queried diet in the few years prior to diagnosis, which may not be the relevant exposure window for PanCa. We also acknowledge the age of these data as a limitation but posit that these associations are relevant on an individual level despite decreasing trends of SSB consumption in recent years on a population level [31]. A strength of our study is that it is one of few studies to look at specific beverage types within the SSB and ASB categories. As a result, this work has the potential improve understanding of the effects of SSB consumption on the underlying biological mechanisms of PanCa. Additionally, our analysis on SSB and ASB consumption and PanCa mortality is novel and provides a basis for future research.

## 5. Conclusions

This study suggests that consuming at least one regular cola per day may be associated with higher odds of PanCa and higher hazard of mortality after PanCa diagnosis. The findings of this study have potential for meaningful public health impact, as PanCa is a deadly malignancy with few established modifiable risk factors and limited treatment options, and cola consumption is a widespread exposure, particularly among Black people, who are also at the greatest risk of PanCa [47,48,49,50,51]. Future work should focus on the role of phosphoric acid in carcinogenesis and the potential synergistic role of phosphoric acid and sugar biologically in PanCa. Epidemiologically, future studies on risk should focus on increasing the sample size and representativeness of diverse groups, including more diet quality factors, and including diabetes status and chronic pancreatitis as covariables. In future prognostic factor research, larger sample sizes are needed to increase statistical power.

## Figures and Tables

**Table 1:** Descriptive characteristics of pancreatic cancer cases and controls from Roswell Park Comprehensive Cancer Center’s Patient Epidemiologic Data System, Buffalo, NY, USA 1982–1998.

Patient Characteristics	Cases (*n* = 213) Mean (±SD) N (%) ¥	Controls (*n* = 852) Mean (±SD) N (%) ¥	*p*-Value *,†
Age at Diagnosis	61.1 (±11.5)	60.8 (±12.1)	0.82
Sex			
Male	115 (54.0)	460 (54.0)	1.00
Female	98 (46.0)	392 (46.0)	
Race			
White	207 (97.2)	793 (93.2)	0.03
Other/Unknown	6 (2.8)	58 (6.8)	
Education			
<High school graduate	52 (24.4)	173 (20.3)	0.21
High school graduate	74 (34.7)	277 (32.5)	
At least some college	87 (40.9)	402 (47.2)	
Income			
<USD 25,000 per year	124 (63.9)	492 (62.2)	0.66
USD 25,000+ per year	70 (36.1)	299 (37.8)	
BMI Categories (kg/m^2^)			
Underweight/Normal Weight (<25.0)	79 (37.1)	350 (41.1)	0.07
Overweight/Obese (≥25.0)	128 (60.1)	494 (58.0)	
Smoking Status			
Never	77 (36.2)	341 (40.0)	0.52
Former	99 (46.5)	375 (44.0)	
Current	37 (17.4)	132 (15.5)	
Pack Years Smoked	24.4 (±27.6)	21.3 (±28.9)	0.17
Family History of PanCa			
Yes	11 (5.2)	22 (2.6)	0.05
No	202 (94.8)	830 (97.4)	
Beverage and Food Consumption			
Regular Soft Drinks			
Never	74 (34.7)	333 (39.1)	0.51
Occasional	49 (23.0)	182 (21.4)	
Habitual	90 (42.3)	337 (39.6)	
All Colas			
Never	78 (36.6)	338 (39.7)	0.65
Occasional	47 (22.1)	189 (22.2)	
Habitual	88 (41.3)	325 (38.2)	
Regular Cola			
Never	104 (48.8)	480 (56.3)	0.10
Occasional	63 (29.6)	231 (27.1)	
Habitual	46 (21.6)	141 (16.6)	
Diet Cola			0.89
Never	144 (67.6)	561 (65.9)	
Occasional	36 (16.9)	151 (17.7)	
Habitual	33 (15.5)	140 (16.4)	
Regular Non-Cola			
Never	130 (61.0)	498 (58.5)	0.73
Occasional	50 (23.5)	222 (26.1)	
Habitual	33 (15.5)	132 (15.5)	
Alcohol SPW	5.1 (±7.9)	5.6 (±8.7)	0.42
Total Vegetable SPW	18.7 (±9.3)	19.9 (±10.8)	0.11
Total Fruit SPW	11.1 (±8.1)	12.1 (±8.4)	0.12
Processed Meat SPW	3.2 (±2.9)	2.8 (±2.9)	0.09

Abbreviations: SD—Standard Deviation, N—Number, SPW—Servings Per Week, PanCa—Pancreatic Cancer. ¥ Total categorical values may not sum to 100% due missing values and/or rounding. † Tested by chi-squared test of differences in proportion. * Tested by Student’s *t*-test for difference in mean.

**Table 2:** Associations between pancreatic cancer and sugary beverage consumption in cases and controls from Roswell Park Comprehensive Cancer Center’s Patient Epidemiologic Data System, Buffalo, NY, USA 1982–1998.

Sugar and Artificially Sweetened Beverages	Number (%) ¥		Age-Adjusted OR		Multivariable-Adjusted OR *	
	Cases	Controls	OR	95% CI	OR	95% CI
Regular Soft Drink Consumption **						
Never	74 (34.7)	333 (39.1)	1.00		1.00	
Occasional (<1 per day)	49 (23.0)	182 (21.4)	1.22	(0.81,1.82)	1.13	(0.75,1.71)
Habitual (1+ per day)	90 (42.3)	337 (39.6)	1.22	(0.86,1.72)	1.15	(0.80,1.65)
All Cola Consumption §						
Never	78 (36.6)	338 (39.7)	1.00		1.00	
Occasional (<1 per day)	47 (22.1)	189 (22.2)	1.10	(0.73,1.65)	1.09	(0.72,1.66)
Habitual (1+ per day)	88 (41.3)	325 (38.2)	1.20	(0.84,1.71)	1.19	(0.82,1.73)
Regular Cola Consumption						
Never	104 (48.8)	480 (56.3)	1.00		1.00	
Occasional (<1 per day)	63 (29.6)	231 (27.1)	1.28	(0.90,1.82)	1.28	(0.88,1.88)
Habitual (1+ per day)	46 (21.6)	141 (16.6)	1.57	(1.05,2.37)	1.55	(1.01,2.39)
Diet Cola Consumption						
Never	144 (67.6)	561 (65.9)	1.00		1.00	
Occasional (<1 per day)	36 (16.9)	151 (17.7)	0.93	(0.62,1.40)	0.96	(0.62,1.49)
Habitual (1+ per day)	33 (15.5)	140 (16.4)	0.92	(0.60,1.41)	0.92	(0.59,1.43)
Regular Non-Cola Soft Drinks						
Never	130 (61.0)	498 (58.5)	1.00		1.00	
Occasional (<1 per day)	50 (23.5)	222 (26.1)	0.86	(0.60,1.24)	0.84	(0.56,1.24)
Habitual (1+ per day)	33 (15.5)	132 (15.5)	0.96	(0.63,1.48)	0.85	(0.54,1.33)

Abbreviations: OR—Odds Ratio, CI—Confidence Interval; * Adjusted for age, sex, smoking status, BMI categories, total vegetable servings per week, processed meat servings per week, and family history of pancreatic cancer; Each beverage type OR was mutually adjusted for the other beverage types in the multivariable analysis; ** Regular Soft Drink Consumption includes regular cola and other regular non-cola soft drinks; § All Cola Consumption includes both regular cola and diet cola; ¥ Total values may sum above or below 100% due to rounding.

**Table 3:** Descriptive characteristics of pancreatic cancer cases from Roswell Park Comprehensive Cancer Center’s Patient Epidemiologic Data System, Buffalo, NY, USA 1982–1998.

Patient Characteristics	Alive ≤ 1 Year (*n* = 133) Mean/Median ^1^ (±SD/IQR) N (%) ¥	Alive > 1 Year (*n* = 80) Mean/Median ^1^ (±SD/IQR) N (%) ¥	*p*-Value *, **, †
Overall Survival (months)	6.1 (±4.4)	23.7 (±29.5)	<0.01
Age at Diagnosis (years)	61.8 (±11.7)	59.8 (±11.0)	0.20
Sex			
Male	75 (56.4)	40 (50.0)	0.37
Female	58 (43.6)	40 (50.0)	
Race			
White	132 (99.3)	75 (93.8)	0.03 **
Other/Unknown	1 (0.8)	5 (6.3)	
Education			
<High school graduate	36 (27.1)	16 (20.0)	0.37
High school graduate	47 (35.3)	27 (33.8)	
At least some college	50 (37.6)	37 (46.3)	
Income			
<USD 25,000 per year	77 (64.2)	47 (63.5)	0.93
USD 25,000+ per year	43 (35.8)	27 (36.5)	
BMI Categories (kg/m^2^)			
Underweight/Normal Weight (<25.0)	45 (33.8)	34 (42.5)	0.30
Overweight/Obese (≥25.0)	83 (62.4)	45 (56.3)	
Smoking Status			
Never	48 (36.1)	29 (36.3)	0.52
Former	59 (44.4)	40 (50.0)	
Current	26 (19.6)	11 (13.8)	
Pack Years Smoked	24.4 (±27.7)	24.3 (±27.5)	0.96
Family History of PanCa	7 (5.3)	4 (5.0)	1.00 **
Beverage and Food Consumption			
Regular Soft Drinks			
Never	47 (35.3)	27 (33.8)	0.28
Occasional	26 (19.6)	23 (28.8)	
Habitual	60 (45.1)	30 (37.5)	
All Colas			
Never	49 (36.8)	29 (36.3)	0.90
Occasional	28 (21.1)	19 (23.8)	
Habitual	56 (42.1)	32 (40.0)	
Regular Cola			
Never	61 (45.9)	43 (53.8)	0.52
Occasional	41 (30.8)	22 (27.5)	
Habitual	31 (23.3)	15 (18.8)	
Diet Cola			
Never	91 (68.4)	53 (66.3)	0.95
Occasional	22 (16.5)	14 (17.5)	
Habitual	20 (15.0)	13 (16.3)	
Regular Non-Cola			
Never	85 (63.9)	45 (56.3)	0.49
Occasional	28 (21.1)	22 (27.5)	
Habitual	20 (15.0)	13 (16.3)	
Alcohol SPW	5.2 (±8.6)	4.9 (±6.6)	0.78
Total Vegetable SPW	17.7 (±9.2)	20.5 (±9.3)	0.03
Total Fruit SPW	10.3 (±8.5)	12.6 (±7.3)	0.05
Processed Meat SPW	3.2 (±3.0)	3.1 (±2.8)	0.72
Clinical Characteristics			
Stage at Diagnosis			
Localized	4 (3.0)	6 (7.5)	<0.01 **
Regional	31 (23.3)	37 (46.3)	
Distant	91 (68.4)	34 (42.5)	
Other/Unknown	7 (5.3)	3 (3.8)	
Histology			
Adenocarcinoma	121 (91.0)	54 (67.5)	<0.01
Other Histologies	12 (9.0)	26 (32.5)	
Treatments Received			
Surgery	18 (13.5)	35 (43.8)	<0.01
Chemotherapy	102 (76.7)	61 (76.3)	0.94
Radiation	39 (29.3)	43 (53.8)	<0.01

Abbreviations: SD—Standard Deviation, IQR—Interquartile Range, N—Number, SPW—Servings Per Week, PanCa—Pancreatic Cancer. ^1^ Median/IQR reported for overall survival; mean/SD reported for all other continuous variables. ¥ Total categorical values may not sum to 100% due missing values and/or rounding. † Tested by chi-squared test of differences in proportion. * Tested by Student’s *t*-test for difference in mean. ** Tested using the Fisher Exact test.

**Table 4:** Associations between pre-diagnostic sugary beverage consumption and mortality after diagnosis of pancreatic cancer from Roswell Park Comprehensive Cancer Center’s Patient Epidemiologic Data System, Buffalo, NY, USA 1982–1998.

Sugar and Artificially Sweetened Beverages	Number (%) ¥		Age-Adjusted HR		Multivariable-Adjusted HR *	
	Dead	Alive	HR	95% CI	HR	95% CI
Regular Soft Drink Consumption **						
Never	69 (34.0)	5 (50.0)	1.00		1.00	
Occasional (<1 per day)	46 (22.7)	3 (30.0)	0.96	(0.66,1.40)	0.83	(0.56,1.26)
Habitual (1+ per day)	88 (43.4)	2 (20.0)	1.33	(0.96,1.84)	1.25	(0.87,1.80)
All Cola Consumption §						
Never	74 (36.5)	4 (40.0)	1.00		1.00	
Occasional (<1 per day)	44 (21.7)	3 (30.0)	0.97	(0.66,1.43)	0.83	(0.54,1.26)
Habitual (1+ per day)	85 (41.9)	3 (30.0)	1.26	(0.91,1.75)	1.28	(0.90,1.81)
Regular Cola Consumption						
Never	97 (47.8)	7 (70.0)	1.00		1.00	
Occasional (<1 per day)	61 (30.1)	2 (20.0)	1.28	(0.92,1.78)	1.06	(0.73,1.53)
Habitual (1+ per day)	45 (22.2)	1 (10.0)	1.43	(0.99,2.07)	1.38	(0.91,2.07)
Diet Cola Consumption						
Never	137 (67.5)	7 (70.0)	1.00		1.00	
Occasional (<1 per day)	35 (17.2)	1 (10.0)	1.13	(0.78,1.64)	1.14	(0.75,1.73)
Habitual (1+ per day)	31 (15.3)	2 (20.0)	1.08	(0.73,1.62)	1.05	(0.70,1.59)
Regular Non-Cola Soft Drink Consumption						
Never	123 (60.6)	7 (70.0)	1.00		1.00	
Occasional (<1 per day)	49 (24.1)	1 (10.0)	0.95	(0.68,1.33)	0.97	(0.66,1.41)
Habitual (1+ per day)	31 (15.3)	2 (20.0)	1.13	(0.76,1.68)	0.93	(0.60,1.46)

Abbreviations: HR—Hazard Ratio, CI—Confidence Interval; * Adjusted for age, sex, smoking status, BMI categories, histology, stage, surgery status, radiation status, and total vegetable servings per week; Each beverage type HR was mutually adjusted for the other beverage types in the multivariable analysis; ** Regular Soft Drink Consumption includes regular cola and other regular non-cola soft drinks; § All Cola Consumption includes both regular cola and diet cola; ¥ Total values may sum above or below 100% due to rounding.

## Data Availability

The data that support the findings of this study are available upon request, but some restrictions apply to the release of these data.

## Supplementary Materials

The following supporting information can be downloaded at: https://www.mdpi.com/article/10.3390/nu15020275/s1, Table S1: Categorization of sugar-sweetened and artificially sweetened beverages from Roswell Park Comprehensive Cancer Center’s Patient Epidemiologic Data System, Buffalo, NY, USA 1982–1998, Table S2: Categorization of consumption patterns of sugar-sweetened and artificially sweetened beverages from Roswell Park Comprehensive Cancer Center’s Patient Epidemiologic Data System, Buffalo, NY, USA 1982–1998, Table S3: Association between pancreatic cancer and regular soft drink, cola full, regular cola, diet cola, and regular non-cola soft drink consumption according to smoking status, BMI, and sex in cases and controls from Roswell Park Comprehensive Cancer Center’s Patient Epidemiologic Data System, Buffalo, NY, USA 1982–1998.
