# Supplementary material for: Sugar Sweetened and Artificially Sweetened Beverage Consumption and Pancreatic Cancer: A Retrospective Study
Source: Nutrients. 2023 Jan 5;15(2):275. doi: 10.3390/nu15020275 (PMC9866356; doi:10.3390/nu15020275)
Supplement: Supplementary file 1 [file nutrients-15-00275-s001.zip › nutrients-2070869-supplementary.pdf]

Table S1. Categorization of sugar-sweetened and artificially sweetened beverages from Roswell Park Comprehensive Cancer Center's Patient Epidemiologic Data System, Buffalo, NY, USA 1982–1998.

	SSB or ASB Category				
	SSB			ASB	Mixed SSB/ASB
	**Regular soft drinks**	**Regular cola**	**Regular non-cola soft drinks**	**Diet cola**	**Cola drinks**
**FFQ Item**					
Regular cola	X	X			X
Diet cola				X	X
Regular non-cola soft drinks	X		X		

Abbreviations: SSB—sugar-sweetened beverages, ASB—artificially sweetened beverages, FFQ—Food frequency questionnaire.

Table S2. Categorization of consumption patterns of sugar-sweetened and artificially sweetened beverages from Roswell Park Comprehensive Cancer Center's Patient Epidemiologic Data System, Buffalo, NY, USA 1982–1998.

	Consumption Category		
**Drinks per day**	**Never**	**Occasional**	**Habitual**
0	X		
<1		X	
1			X
2			X
3			X
4			X
5			X
6			X
>6			X

Table S3. Associaiton between pancreatic cancer and regular soft drink, colla full, regular cola, diet cola, and regular non-cola soft drink consumption according to smoking status, body mass index, and sex in cases and controls from Roswell Park Comprehensive Cancer Center's Patient Epidemiologic Data System, Buffalo, NY, USA 1982–1998.

	Regular Soft Drinks				Cola Full				Regular Cola				Diet Cola				Regular Non-Cola Soft Drinks			
**Stratification Variables**	**Number**		**Multivariable ***		**Number**		**Multivariable ***		**Number**		**Multivariable ***		**Number**		**Multivariable ***		**Number**		**Multivariable ***	
	**Cases**	**Controls**	**OR**	**95% CI**	**Cases**	**Controls**	**OR**	**95% CI**	**Cases**	**Controls**	**OR**	**95% CI**	**Cases**	**Controls**	**OR**	**95% CI**	**Cases**	**Controls**	**OR**	**95% CI**
**<u>Smoking Status</u>**																				
**Never Smoker**																				
Never	32	125	1.00		29	144	1.00		40	196	1.00		48	226	1.00		46	179	1.00	
Occasional (<1 per day)	16	81	0.66	(0.33,1.32)	16	79	1.04	(0.51,2.13)	23	104	1.17	(0.62,2.22)	15	61	1.31	(0.65,2.67)	22	112	0.67	(0.36,1.26)
Habitual (1+ per day)	29	135	0.82	(0.45,1.49)	32	118	1.57	(0.83,2.95)	14	41	2.18	(0.99,4.78)	14	54	1.23	(0.60,2.52)	9	50	0.61	(0.26,1.43)
**Former Smoker**																				
Never	32	153	1.00		37	145	1.00		47	220	1.00		72	231	1.00		63	224	1.00	
Occasional (<1 per day)	27	80	1.48	(0.82,2.68)	26	83	1.22	(0.68,2.20)	31	95	1.52	(0.87,2.65)	16	75	0.69	(0.37,1.30)	22	89	0.85	(0.48,1.51)
Habitual (1+ per day)	40	142	1.32	(0.77,2.27)	36	147	0.95	(0.55,1.63)	21	60	1.64	(0.87,3.08)	11	69	0.54	(0.27,1.10)	14	62	0.76	(0.39,1.50)
**Current Smoker**																				
Never	10	54	1.00		12	48	1.00		17	63	1.00		24	102	1.00		21	93	1.00	
Occasional (<1 per day)	6	21	1.47	(0.44,4.94)	5	26	0.90	(0.26,3.09)	9	31	0.89	(0.28,2.77)	5	14	2.71	(0.71,10.35)	6	20	1.15	(0.35,3.83)
Habitual (1+ per day)	21	57	1.48	(0.53,4.15)	20	58	1.55	(0.59,4.11)	11	38	0.84	(0.28,2.50)	8	16	3.34	(1.12,9.98)	10	19	1.84	(0.66,5.11)
**<u>Body Mass Index</u>**																				
**Normal Weight (BMI 18.5–24.9)**																				
	32	140	1.00		34	155	1.00		43	197	1.00		60	242	1.00		48	208	1.00	
	1	77	0.86	(0.44,1.71)	17	69	1.13	(0.57,2.22)	16	94	0.80	(0.40,1.60)	8	61	0.61	(0.26,1.44)	18	92	1.02	(0.53,1.95)
Habitual (1+ per day)	31	125	1.05	(0.58,1.90)	28	118	1.04	(0.57,1.92)	20	51	1.67	(0.82,3.41)	11	39	1.29	(0.60,2.78)	13	42	1.15	(0.54,2.43)
**Overweight/Obese (BMI ≥ 25.0)**																				
Never	40	186	1.00		42	178	1.00		58	274	1.00		79	310	1.00		77	279	1.00	
Occasional (<1 per day)	31	103	1.33	(0.78,2.27)	28	118	1.06	(0.61,1.84)	45	133	1.71	(1.06,2.76)	28	88	1.19	(0.70,2.01)	32	128	0.74	(0.44,1.22)
Habitual (1+ per day)	57	205	1.26	(0.79,2.02)	58	198	1.36	(0.85,2.19)	25	87	1.51	(0.86,2.66)	21	96	0.89	(0.51,1.54)	19	87	0.71	(0.40,1.27)
**<u>Sex</u>**																				
**Female**																				
Never	38	169	1.00		40	178	1.00		55	248	1.00		62	254	1.00		61	226	1.00	
Occasional (<1 per day)	22	88	0.95	(0.51,1.75)	20	79	1.16	(0.62,2.18)	23	95	1.07	(0.59,1.95)	18	74	1.18	(0.62,2.24)	22	116	0.65	(0.36,1.17)
Habitual (1+ per day)	38	13	1.09	(0.63,1.88)	38	135	1.28	(0.73,2.24)	20	49	1.92	(0.98,3.77)	18	64	1.14	(0.61,2.14)	15	50	0.87	(0.44,1.74)
**Male**																				
Never	36	164	1.00		38	160	1.00		49	232	1.00		82	307	1.00		69	272	1.00	
Occasional (<1 per day)	27	94	1.19	(0.67,2.12)	27	110	1.01	(0.57,1.79)	40	136	1.38	(0.83,2.30)	18	77	0.84	(0.46,1.53)	28	106	1.02	(0.60,1.74)
Habitual (1+ per day)	52	202	1.17	(0.72,1.91)	50	190	1.12	(0.68,1.85)	26	92	1.37	(0.78,2.43)	15	76	0.79	(0.42,1.49)	18	82	0.86	(0.47,1.57)

Abbreviations: BMI—body mass index, OR—odds ratio, CI—Confidence interval. * Models adjusted for age, sex, BMI categories, smoking status, total vegetable servings per week, processed meat servings per week, family history of pancreatic cancer, and mutual adjustment for the other beverage type consumption. Stratified models exclude the stratifying variable from the model.
